# COVID Feel Good—An Easy Self-Help Virtual Reality Protocol to Overcome the Psychological Burden of Coronavirus

**DOI:** 10.3389/fpsyt.2020.563319

**Published:** 2020-09-23

**Authors:** Giuseppe Riva, Luca Bernardelli, Matthew H. E. M. Browning, Gianluca Castelnuovo, Silvia Cavedoni, Alice Chirico, Pietro Cipresso, Dirce Maria Bengel de Paula, Daniele Di Lernia, Javier Fernández-Álvarez, Natàlia Figueras-Puigderrajols, Kei Fuji, Andrea Gaggioli, Jose Gutiérrez-Maldonado, Upyong Hong, Valentina Mancuso, Milena Mazzeo, Enrico Molinari, Luciana F. Moretti, Angelica B. Ortiz de Gortari, Francesco Pagnini, Elisa Pedroli, Claudia Repetto, Francesca Sforza, Chiara Stramba-Badiale, Cosimo Tuena, Clelia Malighetti, Daniela Villani, Brenda K. Wiederhold

**Affiliations:** ^1^IRCCS Istituto Auxologico Italiano, Milan, Italy; ^2^Department of Psychology, Università Cattolica del Sacro Cuore, Milan, Italy; ^3^Become-Hub, Milan, Italy; ^4^Virtual Reality and Nature Lab, Clemson University, Clemson, SC, United States; ^5^Sociedad Española de Realidad Virtual y Psicología, Las Rozas – Madrid, Spain; ^6^Department of Clinical Psychology and Psychobiology, University of Barcelona, Barcelona, Spain; ^7^Division of Psychology, University of Tsukuba, Tokyo, Japan; ^8^Department of Media and Communication, Konkuk University, Seoul, South Korea; ^9^The Centre for the Science of Learning & Technology (SLATE), University of Bergen, Bergen, Norway; ^10^Psychology and Neuroscience of Cognition Research Unit, University of Liège, Liège, Belgium; ^11^Faculty of Psychology, University of eCampus, Novedrate, Italy; ^12^Virtual Reality Medical Center, La Jolla, CA, United States; ^13^Virtual Reality Medical Institute, Brussels, Belgium

**Keywords:** COVID, virtual reality, self-help, stress, emotion regulation, mental health

## Abstract

**Background:**

Living in the time of the COVID-19 means experiencing not only a global health emergency but also extreme psychological stress with potential emotional side effects such as sadness, grief, irritability, and mood swings. Crucially, lockdown and confinement measures isolate people who become the first and the only ones in charge of their own mental health: people are left alone facing a novel and potentially lethal situation, and, at the same time, they need to develop adaptive strategies to face it, at home. In this view, easy-to-use, inexpensive, and scientifically validated self-help solutions aiming to reduce the psychological burden of coronavirus are extremely necessary.

**Aims:**

This pragmatic trial aims to provide the evidence that a weekly self-help virtual reality (VR) protocol can help overcome the psychological burden of the Coronavirus by relieving anxiety, improving well-being, and reinforcing social connectedness. The protocol will be based on the “Secret Garden” 360 VR video online (www.covidfeelgood.com) which simulates a natural environment aiming to promote relaxation and self-reflection. Three hundred sixty–degree or spherical videos allow the user to control the viewing direction. In this way, the user can explore the content from any angle like a panorama and experience presence and immersion. The “Secret Garden” video is combined with daily exercises that are designed to be experienced with another person (not necessarily physically together), to facilitate a process of critical examination and eventual revision of core assumptions and beliefs related to personal identity, relationships, and goals.

**Methods:**

This is a multicentric, pragmatic pilot randomized controlled trial involving individuals who experienced the COVID-19 pandemic and underwent a lockdown and quarantine procedures. The trial is approved by the Ethics Committee of the Istituto Auxologico Italiano. Each research group in all the countries joining the pragmatic trial, aims at enrolling at least 30 individuals in the experimental group experiencing the self-help protocol, and 30 in the control group, over a period of 3 months to verify the feasibility of the intervention.

**Conclusion:**

The goal of this protocol is for VR to become the “surgical mask” of mental health treatment. Although surgical masks do not provide the wearer with a reliable level of protection against the coronavirus compared with FFP2 or FFP3 masks, surgical masks are very effective in protecting others from the wearer’s respiratory emissions. The goal of the VR protocol is the same: not necessarily to solve complex mental health problems but rather to improve well-being and preserve social connectedness through the beneficial social effects generated by positive emotions.

## Introduction

### Background

Living in the time of the COVID-19 means experiencing not only a global health emergency but also extreme psychological stress that puts a strain on our identity and our relationships. Coronavirus and the associated isolation and quarantine require people to manage three different psychological dilemmas simultaneously (1).

- the stress due to the disease,
- the inaccessibility to physical places,
- and the sense of community crisis.

The core stress of the disease comes from the worry and concerns about personal health and the health of friends and family members. This stress can be exacerbated both among general public and medical staff *via* the vicarious traumatization effect (2) when empathizing with those suffering, resulting in fatigue, physical decline, sleep disorder, irritability, inattention, fear, and despair (3).

The traumatic effects are further aggravated by living in quarantine and its restrictions on movement and social interaction. In fact, evidence has shown that quarantine causes significant psychological effects including post-traumatic stress symptoms, confusion, and anger (4).

The inaccessibility to physical places is one of the first clear negative effects of quarantine. A conflict arises that is provoked by losing access to physical places where people can meet and that we feel belongs to us. A “place” can be understood as any space delimited by borders and that gives identity to individuals and represents a space to be. Related to place is the concept of place attachment (5) which is the bonding of people to places. This bond includes cognitive and emotional components and is a common phenomenon observed across cultures with significant psychological benefits (6). However, quarantine disrupts place attachment, and therefore, has negative implications. As noted by Scannel and Gilford (6), separation from one’s significant place can be devastating: “broken or stretched place bonds are associated with physical health problems, lower grades, sadness, longing alienation, and disorientation” (pp. 256–257). Women tend to report stronger place attachment than men (6) and, therefore, the disruption of place attachment provoked by the quarantine may have stronger psychological effects in women.

Å crisis of the sense of community is caused by disconnect from the places where communities are born (7), and provide significant negative effect on subjective well-being (8, 9). The disruption of places produced by the quarantine also affects the communities that use these places to meet and interact. Without everyday places to meet at—such as the workplace and the classroom—friends and acquaintances are more difficult to reach and to interact with. This weakens social bonds and declines the social significance of the local community in terms of social capital and interpersonal support.

These negative psychological effects may be aggravated by other stressors such as having inadequate basic supplies (e.g., food, etc.), insufficient clear guidelines about actions to take and the prolonged duration of quarantine, the interruption of professional activities and the subsequent financial loss (4).

In this view, any strategy that aims to reduce the psychological burden of coronavirus is extremely necessary (10). As recently underlined by Holmes and colleagues (10): “There is an urgent need for the discovery, evaluation, and refinement of mechanistically driven interventions to address the psychological, social, and neuroscientific aspects of this pandemic. This includes bespoke psychological interventions to boost wellbeing and minimize mental health risks across society.” (p. 10). Crucially, given the mandatory loneliness resulting from lockdown measures, easy-to-use, inexpensive, and scientifically validated self-help solutions could be the key (11–16).

### Aims

This pragmatic trial seeks to provide the evidence that a weekly self-help protocol based on a virtual reality experience—“The Secret Garden”, available in the www.covidfeelgood.com website—can help to overcome the psychological burden of the Coronavirus.

It is important to underline that the goal of the self-help protocol is not to provide a full structured psychological intervention, but to build the “surgical mask” of mental health support. Surgical Masks do not provide the wearer with a reliable level of protection against coronavirus (20%) versus the 95/99% of FFP2 and FFP3 masks. However, they are very effective in protecting others from the wearer’s respiratory emissions, and their use is significantly better than wearing a scarf.

The self-help VR protocol assessed in the trial aims to do the same. The goal is not to solve complex mental health problems, but rather to reduce the burden of the coronavirus: Specifically, the protocol aims at relieving anxiety and stress and improving well-being and social connectedness through these two assets:

- The potential of (also) simulated nature for improving people’ wellbeing, health and ameliorating anxiety and depressive feelings (17–19) with or without a direct interaction with it (20). Crucially, simulated nature can ameliorate negative moods in the short-term, and besides individual preferences towards nature (20);
- The potential of all types of VR formats, including 360° videos, to resemble even distant, complex, even paradoxical scenarios in a realistic, immersive and engaging way, thus providing the illusion of being really “there”, in the simulated place (21–23).

This last asset provided by VR is the pathway to the *transformation* of people’s experiences in a several and profound ways (24–26). For instance, immersive experiences can enhance individuals’ personal efficacy and self-reflectiveness through the manipulation of the sense of presence, flow, and emotional engagement (27, 28). Moreover, VR’s unique ability to evoke complex emotions, which are drivers of people’ health, wellbeing and sense of social connection (29, 30), would allow designing unique experiences leading to long-terms benefits.

The protocol will be based on the same 10-min 360° VR video (“The Secret Garden”) used by Chirico and colleagues (29, 30).

**Figure 1:**
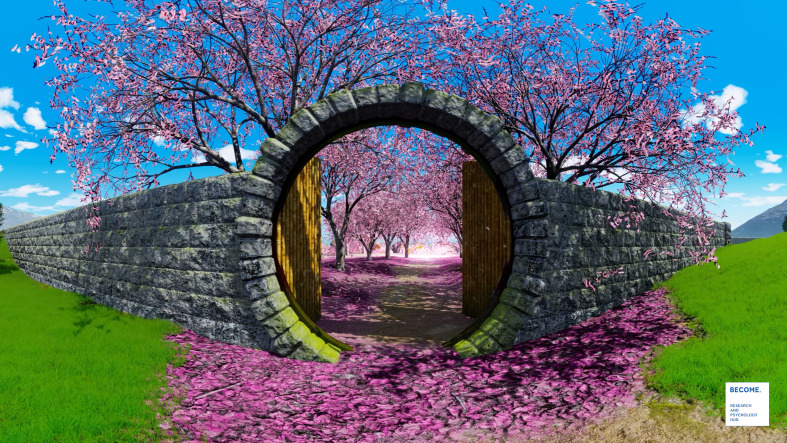
A screenshot of the “Secret Garden” VR experience.

“The Secret Garden” VR video has been developed through an integrated process involving psychologists, 3D artists, musicians, storytellers and designers (**Figure 1**). This immersive experience storyboard has been:

- written by well-being psychologists to mimic the structure and the experience of walking in a Japanese garden (31) providing the visual (i.e., the flow of water) and auditory (i.e., the sound of running water) natural elements available outdoors.
- converted in a VR experience by 3D specialists using the Unreal Engine 4 technology.
- dubbed by a professional dubber in the different languages used in the trial using the back-translation method. In all languages a a slow, calm, clear voice provides a relaxation induction structured following the principles of Compassion Focused Therapy (32, 33). Specifically, the induction aims at deactivating the human threat protection system and activating the soothing system (with a mindset attended to giving and receiving care, affecting, and nurturance).

We decided to use a computer-graphic 360° video (artificial) instead of a video-recorded format (natural) for the following reasons. First, we selected a video whose efficacy in positive emotional induction was already validated in a previous study. Second, using computer-graphic it is easier to manipulate specific features of the natural environment aimed at improving positive affect (i.e., the extreme blossoming of the peach trees presented in the VR experience) that are more difficult to achieve using a real natural environment. Third, during lockdown was impossible access to real natural places.

Three hundred sixty–degree videos have the power to virtually transport users, immersing them in the video recording, allowing them to actively explore its content and experience the video from any angle. With this regard, as shown by Robertson and colleagues the neural representations of the part of the 360° video presented in VR (the scene within the current field of view) prime the associated representations of the full panoramic environment (34). In other words, 360° videos generate a dynamic interplay between memory and perception that can be used to improve the features of these cognitive processes and to update their content.

To anchor the generated update to the autobiographical memory of the user, at end of the VR exposure the subjects will be asked to perform together different tasks related to personal identity and interpersonal relationships (35). These tasks, are an adaptation of the different “emotional prescriptions” designed by the psychologist Guy Winch (35) to react to personal experiences that generate emotional pain: loneliness, rejection, or rumination. The tasks want to achieve the following goals: a) to pay attention and recognize emotional pain; b) work to treat it before it feels all-encompassing; c) monitor and protect self-esteem; d) find meaning even in difficult times. The full description of the tasks is provided in **Table 1**.

**Table 1:** Descriptions of the daily exercises.

**Day 1: Fight Rumination** - **The Problem:** Reflecting on the coronavirus and its consequences and dwelling on them in one’s mind is natural. However, to prevent them from becoming a fixation, one must learn to control them. - **The Goal:** To do this, start by changing your point of view. For example, try to imagine that you are a different person—a doctor who has to treat a patient, a politician who has to decide what to do, a nurse who has to support the patient in the last moments of life—and describe in writing the emotions that occur and what you would do. Then, try to describe in writing how you would vent the anger, feelings of helplessness, and/or other difficult emotions these situations can generate. - **Social Experience:** If you want, you can discuss your feelings with your partner and compare them with his/hers to understand the similarities and differences. **Day 2: Awaken your Self Esteem** - **The Problem:** Quarantine, by forcing us to always repeat the same things with the same people in the same physical space, can make us apathetic and reduce our self-esteem. - **The Goal:** To awaken it, list in writing the five aspects of your character and your personality that you own and appreciate, put them in order of importance, and discuss the following two points for each: why is it important and how does it influence your life and relationships? - **Social Experience:** If you want, you can discuss them with your partner and check whether he/she shares the same vision or not and why. **Day 3: Awaken your Autobiographical Memory** - **The Problem:** The lack of places weakens our autobiographical memory, leading us to remember always the same days and making us lose the memory of who we are and what we want. - **The Goal:** To awaken it, list in writing four moments and/or events in your life that have helped you to be who you are and a moment of the coronavirus emergency that you particularly remember. For each, discuss the following points: why are they important, what emotions did they elicit in me, and when have I experienced similar emotions? - **Social Experience:** If you want, you can discuss them with your partner and compare them with his/hers to understand similarities and differences. **Day 4. Awaken your Sense of Community** - **The Problem:** The weakening of the sense of community can increase our sense of loneliness. - **The Goal:** To awaken the sense of community, list the five most significant people in your relationships. For each, discuss the following points: why are they important, are you also important to them, and why? - **Social Experience:** If you want, you can discuss them with your partner and compare them with his/her choices to understand similarities and differences. **Day 5. Awaken your Goals and/or Dreams** - **The Problem:** The continuous sense of anxiety generated by the coronavirus emergency can lead to the halting of our daily activities, making us lose sight of our goals and aspirations. - **The Goal:** To awaken them, list in writing three concrete goals and two dreams/aspirations that you would like to achieve after the quarantine. For each, discuss the following points: why are they important to you, what do you miss to reach them, and what can you do now? - **Social Experience:** If you want, you can discuss them with your partner to understand similarities and differences. **Day 6. Boost your Empathy** - **The Problem:** All relationships always involve a giving and receiving. But to effectively “give” we must be able to “receive” the other’s point of view. - **The Goal:** To do this, think about the last significant interaction you had with each of the five people you indicated on day 4 and try to describe in writing the emotions that you think they felt at that time. - **Social Experience:** Again, you can discuss your emotions with your partner and compare them to understand similarities and differences. **Day 7. Plan your change** - **The Problem:** Coronavirus, willingly or unwillingly, forces us to change and manage new situations such as quarantine, close coexistence with children and spouse, lack of relationships, and so on. - **The Goal:** You can try using this period to try to improve your life. Start by identifying in writing three aspects of your life with which you are dissatisfied. Then, on a first sheet describe the possible solutions by placing them in order of probability of success and cost/opportunity. On a second sheet, identify potential problems and their impact. Finally, on the third sheet, identify the tools and/or information that you are lacking but which can help you reach the possible solutions. - **Social Experience:** Finally, tear off the problems sheet and use the other two sheets to plan strategies that can move you closer to solving your problems with the support of your partner.

### Hypotheses

The study has the following hypotheses:

1. The use of the weekly VR self-help protocol will reduce the level of depression, anxiety, perceived stress, and hopelessness;
2. The use of the weekly VR self-help protocol will promote the relaxation and social connectedness of the participants.

## Methods and Analysis

### Study Design

This will be a multicentric pragmatic pilot randomized controlled trial involving individuals who have experienced the isolation and quarantine associated to the Coronavirus pandemic. In contrast to explanatory trials that often include highly selected, “ideal” patients, pragmatic trials adopt broader eligibility criteria that reflect the diversity of patients who are treated in routine situations (36). In accordance with the real-life approach of our study, we expect a heterogeneous patient population, which is the goal of pragmatic trials. The trial overall will show results that pertain to the heterogeneous population, including subgroups representative of the target population. Potential participants will be contacted through web, e-mail, or social media postings. Individuals who will express interest in participating to the trials will be contacted to verify if they meet the inclusion criteria (below). Each eligible participant will provide written informed consent for study participation.

After signing the informed consent, participants will be randomly assigned to the experimental (VR) or control (Waiting-list) conditions (Two-Group Random Assignment Pretest–Posttest Design). Baseline measures of anxiety, depression, perceived stress, general wellbeing, and relaxation will be collected at the baseline (Day 7), before the starting of the protocol (Day 0), at the end of the protocol (Day 7) and after a 2-week follow-up (Day 21). State measures of anxiety, perceived stress, and relaxation will be collected each day of the protocol after the experimental condition, from Day 1 to Day 7.

### Randomization

Randomization will be done by a computer algorithm written in SAS (37). Participants will be randomly allocated in a 1:1 ratio, and using randomly chosen block sizes (37).

### Sample

Each research group in all the countries joining the trial (at the moment Italy, Spain, and USA) will recruit two samples of at least 30 subjects. The experimental group will experience the VR protocol described below.

Inclusion criteria will be:

- adult patients (≥18 years);
- of mother tongue of the country where they will be enrolled;
- have experienced at least two months of quarantine or isolation related to the coronavirus pandemic;
- give full, written, informed consent;
- have the availability of a smartphone and a cardboard VR headset;
- availability and agreement of a partner for conducting the self-help component of the treatment.

To reflect routine, everyday practice, subjects will not excluded if they have other medical conditions, or are taking medication (38).

Exclusion criteria, assessed through an interview with the participants will be:

- Visual of ear impairments that can limit the participation to the protocol.
- Participants reporting vestibular and/or balance disorders.

### Psychological Measures

Participants will complete two series of questionnaires.

The ***baseline*, *post intervention*, *and follow-up measures*** are a series of semi-trait questionnaires that will assess how the participants felt in the previous week. These instruments will assess perceived stress, depression and anxiety, hopelessness, social connectedness, fear of coronavirus, and social contacts interactions. The compilation will require approximately 20 min.

The ***state measures*** are a series of questionnaires and scales that assess how participants feel after the experimental procedure. They will be collected daily (from Day 1 to Day 7) immediately after the protocol and will assess state anxiety, self-reported stress, and relaxation. The compilation will require approximately 5–10 min.

#### Baseline, Post Intervention, and Follow Up Measures Collected at Day 0 and at Day 7

- **Perceived Stress Scale (PSS):** The PSS (39) is a widely used instrument for measuring individuals’ perceived stress. It assesses how much our daily situations are perceived as stressful, unpredictable, and ultimately overloading. Moreover, the PSS also assess the current level of perceived stress directly, providing a reliable and robust instrument for stress assessment. Items in the PSS assess feelings and thoughts the last month, however the scale will be adapted to assess perceived stress in the last week. The instrument is composed by 10 items on a 5-point Likert A composite score of the 10 items provide a general measure of perceived stress.
- **Depression Anxiety Stress Scale (DASS-21):** The DASS-21 (40) is a short version of the original instrument developed by Lovibond and it is composed of 21 items divided into 3 subscales that measure anxiety, depression, and perceived stress. The scale assesses how the participants felt in the previous 7 days on a 4-point Likert. Scores are computed individually for each subscale. A composite score of general distress is obtained by computing all the three subscale scores together.
- **Beck Hopelessness Scale (BHS):** The BHS (41) is a self-report instrument that measures pessimistic tendencies or negative attitude towards the future within three major aspects of hopelessness: feelings about the future, loss of motivation, and expectations. The scale is composed of 20 items with a True/False response.
- **Social Connectedness Scale (SCS):** The SCS (42) is composed of 8-items and aims at measuring how much the individual feels connected to other persons or to the social context. The scale asks to evaluate agreement or disagreement to several contextual statements on a 6-point Likert scale. Higher scores represent a higher sense of social connectedness.
- **Fear of Coronavirus (FCOR):** FCOR is a series of statements presented in (43) to measure the level of fear toward the COVID-19 pandemic. FCOR is composed of 8 items that explore different components of fear such as the personal experience of concern regarding the current situation, avoidance behaviors and attention bias. Each statement is evaluated on a 5-point Likert.
- **Online and offline contact (COO):** COO is a series of questions (44) to measure the number and quality of online and offline contacts during the COVID-19 pandemic. Individuals are asked to report the number of online contacts in the past week evaluating on a 5-point likert scale how close they felt to those contacts. The same two questions are repeated for offline contacts.

#### State Measures Collected From Day 1 to Day 7

- **Smith Relaxation State Inventory 3 (SRSI3):** The SRSI3 (45) is the revised form of the original Smith Relaxation State inventory and it measures both relaxation and perceived stresses. Individuals rate their agreement to several statements on a 6-point Likert scale. The scale is composed of 38 items; however, it is divided into several subscales that can be selected independently. For this protocol, the following subscales have been selected, for a total of 20 items: rest/refresh, energized, physical relaxation, at ease/peace, joy, mental quiet, aware, somatic stress, emotional stress, and cognitive stress.
- **Subjective Units of Distress Scale (SUDS):** The SUDS (46) is simple numeric rating scale from 0 to 100 that measures the level of distress perceived by the individual. It is a reliable measure of state distress, commonly used in cognitive behavioral therapy.

#### Post Intervention Measure Collected at Day 7

- **Negative Effects Questionnaire (NEQ):** The self-report measure consists of three parts for a total of 32 items (47). First, respondents endorse specific items in case they have occurred or not during treatment, yes/no (dummy coded as a variable: 1/0). Second, the respondents rate how negatively the negative effect was on four-point Likert-scale, ranging from “Not at all” to “Extremely”, 0–4 (“0” being minimum and “4” being maximum). Third, the respondents attribute the negative effect to “The treatment I received” (1) or “Other circumstances” (0) (dummy coded as a variable: 1/0).
- **Simulation Sickness Questionnaire (SSQ):** The self-report measure is composed by 16 items used to users’ level of sickness symptoms after a VR experience (48).
- **Final Interview:** This final interview aims at collecting any additional information related to practical challenges of using the VR app and coordinating the self-help social task.

### Study Period

The enrolment is planned to start from June 2020 and will last until the planned number of enrolled patients has been met.

### Outcomes

Considering the presented hypotheses, the primary outcomes expected for the group that will perform the experimental VR procedure compared to the control group are:

- A reduction in anxiety, depression, perceived stress, and hopelessness, as measured by DASS-21, PSS, and BHS.
- A reduction in state anxiety and subjective distress, as measured by SUDS.
- And an increase in relaxation, as measured by SRSI3.

Secondary outcomes of the protocol are

- a decrease in fear of the coronavirus, as measured by FCOR;
- an increase in social connectedness, as measured by SCS;
- an increase in feelings of closeness to online and offline contacts, as measured by COO.

### Description of the Intervention

The 10-min “Secret Garden” 360° VR experience available on the www.covidfeelgood.com website will be used for one week, once per day. To experience the “Secret Garden” the sample will need:

- any Android of iOS smartphone with installed the YouTube App;
- any VR headset compatible with the Cardboard format. These headsets are easily available in online stores for a price ranging between 10 and 50 US$.

Each individual will involve a partner in the process who will share the VR exposure, to discuss the emotions and reflections induced by it. Specifically, at the end of the VR exposure the subjects will be asked to perform together different tasks related to personal identity and interpersonal relationships (35). The full description of the tasks is provided in **Table 1**.

## Statistical Analysis

Categorical variables will be compared using Fisher or chi-square tests and continuous variables using t test or Mann-Whitney tests, as appropriate. Groups will be compared for variables such as sex, age, education, geographic area, stage of disease, type of proposed treatment, and other available data. To assess the effectiveness of the intervention, groups will be compared with a 2 × 2 repeated measure mixed ANOVA for the pre and post measures (factor Group X factor Time: pre and post). Analysis will be performed for all the relevant variables: perceive stress, anxiety, depression, hopelessness, fear of the coronavirus, and social contacts. A repeated measures ANOVA (factor Group × factor Time: day of the week) will compare treatment effects within the seven-day intervention, for all the relevant measures: relaxation, perceived stress, and state anxiety. Tests of statistical significance and confidence intervals will be two-sided; a p < 0.05 will be considered to be statistically significant. Statistical computations and data analysis will be performed using R, a multi-platform (Windows, UNIX, Mac OS), free software environment for statistical computing and graphics.

## Power Size Calculation

Power size calculation was performed with GPower 3.1. Considering an anticipated effect size (f) of.25, an alpha set at.05, 2 groups, and a.95 statistical power, the total sample size required is N = 54.

## Discussion

Living in the time of the coronavirus means experiencing not only a global health emergency but also extreme psychological stress that puts a strain on our identity and our relationships. The fears about personal health and the health of friends and family members, and the effects of the quarantine generate significant psychological effects including post-traumatic stress symptoms, depression confusion, and anger. These negative psychological effects may be enhanced by other stressors such as having inadequate basic supplies (i.e., food, masks, etc.), insufficient clear guidelines about actions to take and the duration of quarantine, the interruption of professional activities and the related financial loss (4). In this view, any strategy that aims to reduce the psychological burden of the coronavirus is extremely necessary (10). In particular, the outbreak of coronavirus is rapidly changing stakeholders’ attitudes towards e-mental health, and this should be harnessed given the fact that many technological solutions are not only cost-effective but nowadays the only possible intervention that confined individuals can receive (49). Despite the undoubted negative consequences of this context, it can be also conceived as an opportunity to achieve *an implementable revolution in digital mental health* (50).

This pragmatic pilot trial seeks to understand if and how a weekly self-help protocol—The Secret Garden—can help overcome the psychological burden of the coronavirus. To reach this goal, the protocol will use virtual reality (24) to provide a transformative experience (27, 51, 52) by offering a natural digital place in which subjects can relax and reflect. This effect will be enhanced by different daily social tasks aiming at facilitating a process of critical examination and eventually revision of core assumptions and beliefs. It is important to underline that the goal of the self-help protocol is not to provide a full structured psychological intervention, but to build the “surgical mask” of mental health support. Its goal is not to solve complex mental health problems, but rather to reduce the coronavirus of the by relieving anxiety and stress and improving interpersonal relationships: when a user is positive and healthy, he/she generates a positive social effect that contributes to the well-being of the community. If the present pragmatic pilot trial will support the feasibility of the approach, further actions to promote the dissemination and the use of the self-help protocol will be encouraged.

## Ethics Statement

The trial is approved by the Ethics Committee of the Istituto Auxologico Italiano.

## Author Contributions

GR conceived the original idea, designed the study and the original protocol. GR, AC, AO, DL, BW and MB wrote the paper. LB and FS developed the “Secret Garden” VR environment used in the study. BW supervised the clinical framework of the study. BW, JG-M, AO, LM, NF-P, MM, KF, DP, and UH contributed to the translations of the protocol in different languages. All authors contributed to the article and approved the submitted version.

## Funding

This paper was supported by the Marie Skłodowska-Curie Innovative Training Networks AffecTech (project ID:722022 - https://www.affectech.org/) and Entwine (project ID: 814072 - https://entwine-itn.eu/) funded by the European Commission H2020.

## Conflict of Interest

The authors declare that the research was conducted in the absence of any commercial or financial relationships that could be construed as a potential conflict of interest.

The reviewer SV declared a past co-authorship with one of the authors GR to the handling Editor.
